# Editorial: Overeating and Decision Making Vulnerabilities

**DOI:** 10.3389/fpsyg.2019.00587

**Published:** 2019-03-22

**Authors:** Qinghua He, Xiao Gao, Yonghui Li, Hong Chen

**Affiliations:** ^1^Faculty of Psychology, Southwest University, Chongqing, China; ^2^Key Laboratory of Cognition and Personality, Ministry of Education, Southwest University, Chongqing, China; ^3^Key Laboratory of Mental Health, Institute of Psychology, Chinese Academy of Sciences, Beijing, China; ^4^Chongqing Collaborative Innovation Center for Brain Science, Chongqing, China; ^5^Southwest University Branch, Collaborative Innovation Center of Assessment Toward Basic Education Quality at Beijing Normal University, Chongqing, China

**Keywords:** overeating, decision making, obesity, binge eating, anorexia nervosa

Overweight and obesity are rapidly becoming a central public health challenge around the world (He et al., 2014a,b; He et al., 2015). For example, in the United States, nearly 65 % of adults are overweight or obese (Stein and Colditz, 2004). Overweight and obesity are associated with increased risk for cardiovascular/metabolic diseases, as well as several common adult cancers (Renehan et al., 2008). Because the fundamental cause of overweight and obesity is an energy imbalance between calories consumed and calories expended, the solution to this problem appears very simple: eat in moderation and engage in regular physical activity. However, this commonsense advice is difficult to follow for many people.

There is mounting evidence that the inability to resist calorie-rich and highly appetitive food represents a special case of addiction behavior (Chen et al.; Kelley and Berridge, 2002; Rolls, 2007; Trinko et al., 2007; Volkow et al., 2008). Similar to other drug addicts, poor decision making and impulse control may facilitate overeating, especially when faced with a constant supply of highly palatable food. This Research Topic aimed to gather a group of articles discussing the relationship between eating and decision making, including eating disorders like bulimia and anorexia.

With this scope, this research topic have assembled articles from a number of scientists who have made important contributions to this evolving field, including two Hypothesis and Theory articles (Chen et al.; Zhang and Coppin), one Protocol (Brevers et al.), and 7 original researches (Chen et al.; Lehner et al.; Li et al.; Lyu et al.; Vicario and Felmingham; Yan et al.; Zhang et al.).

## Hypothesis and Theory articles

We proposed a model of triadic neural systems for problematic eating in this research topic (Chen et al.). This model includes (a) a reward anticipation and processing system; (b) a reflective and inhibitory control system; and (c) an interoceptive awareness system. This model utilized similar tripartite neural models of Internet Gaming Disorder (Wei et al., 2017), pathological gambling and addiction (Noël et al., 2013a,b), and it had been demonstrated using fMRI techniques with dynamic causal modeling (He et al., 2019).

Zhang and Coppin analyzed the importance of memory in food valuation and choices in obese individuals (Zhang and Coppin). They described converging evidence on different forms of memory impairments accompanying obesity. Building on these findings, they formulate a general neuropsychological framework and discuss how dysfunctions in the formation and retrieval of memory may interfere with adaptive decision making for food.

## Protocol

Brevers and colleagues proposed a research protocol aimed to explore the use of a mobile-phone application in treatment for obesity (Brevers et al.). This study protocol will run for 2 years with smartphone application collecting the 4 weeks use data, self-report measures, and participants' feedbacks.

## Original researches

The 7 original research articles provided various angles for overeating and decision making vulnerabilities, including structural MRI (Zhang et al.), functional MRI (Chen et al.), eye movement (Lehner et al.), artificial intelligence (Li et al.), and survey/behavior assessment of patients (Lyu et al.; Vicario and Felmingham; Yan et al.).

Using structural MRI and voxel-based morphometry (VBM) method, Zhang et al. investigated the difference of gray matter volume (GMV) between obese participants and controls. Results suggested that obese men only showed a significantly increased GMV in the left putamen. Further analysis suggested that the GMV of left putamen could predict the BMI and insulin level.

Using functional MRI, Chen et al. measured brain activity of undergraduate young females when they performing a food rating task. They rated various kinds of food on their taste, healthy, and willingness to eat. Behavioral results showed a positive correlation between taste rating and willingness to eat, a negative correlation between healthy rating and emotional eating, as well as a positive correlation between taste rating and external eating. MRI data suggested that activity in DLPFC were positively correlated with successful self-control; and activity in midcingulate cortex was positively correlated with failed self-control.

Lehner et al. investigated the group difference of eye movement during Pavlovian conditioning to measure the incentive salience amongst normal-weight, overweight, and obese individuals (Lehner et al.). Results showed that the goal-directed behavior of overweight individuals was more strongly influenced by food-predicting cues than that of normal-weight and obese individuals. The fixation style also exhibited a complex interaction with the weight category.

Li et al. proposed an intelligent recommendation techniques for consumers' food choices in restaurants (Li et al.). Results suggested that this artificial intelligence based technique can provide effective dish recommendation for customers. This system could be used to aid food choices for obese individuals.

The study by Lyu et al. hypothesized that women with binge eating would show greater deficits in response inhibition than control group tested by flanker task (Lyu et al.). Results suggested that they responded slower for incongruent trials than congruent trials, while no difference were detected for controls.

Similarly, Yan et al. investigated the associations between decision-coping patterns, monetary decision-making, and binge-eating behavior in a large sample of college students (Yan et al.). Results suggested that, compared with the non-binge-eating group, the binge-eating group displayed elevated scores on maladaptive decision-making patterns.

Lastly, study by Vicario and Felmingham investigated the time perception in adolescent with anorexia nervosa (Vicario and Felmingham). Results suggested patients with anorexia nervosa displayed lower timing accuracy than controls.

The wealth of theories, protocol, and original researches covered by the authors in this research topic uncovered the basic underlying mechanism for overeating and decision making vulnerabilities. We hope to provide some insights for weight management, treatment of obesity and related disorders through this window.

## Author Contributions

QH and XG wrote the first draft. YL and HC made critical revision. QH, XG, YL, and HC approved the final version of the manuscript.

### Conflict of Interest Statement

The authors declare that the research was conducted in the absence of any commercial or financial relationships that could be construed as a potential conflict of interest.
